# Towards Secure, Decentralised, and Privacy Friendly Forensic Analysis of Vehicular Data

**DOI:** 10.3390/s21216981

**Published:** 2021-10-21

**Authors:** Lydia Negka, Georgios Spathoulas

**Affiliations:** 1 Department of Computer Science and Biomedical Informatics, University of Thessaly, 35131 Lamia, Greece; lnegka@uth.gr; 2 Department of Information Security and Communication Technology, Norwegian University of Science and Technology (NTNU), NO-2802 Gjovik, Norway

**Keywords:** forensics, vehicular, blockchain, integrity, automation, privacy, traffic, simulation

## Abstract

The automotive industry has been transformed through technological progress during the past decade. Vehicles are equipped with multiple computing devices that offer safety, driving assistance, or multimedia services. Despite these advancements, when an incident occurs, such as a car crash, the involved parties often do not take advantage of the technological capabilities of modern vehicles and attempt to assign liability for the incident to a specific vehicle based upon witness statements. In this paper, we propose a secure, decentralized, blockchain-based platform that can be employed to store encrypted position and velocity values for vehicles in a smart city environment. Such data can be decrypted when the need arises, either through the vehicle driver’s consent or through the consensus of different authorities. The proposed platform also offers an automated way to resolve disputes between involved parties. A simulation has been conducted upon a mobility traffic dataset for a typical day in the city of Cologne to assess the applicability of the proposed methodology to real-world scenarios and the infrastructure requirements that such an application would have.

## 1. Introduction

Technology advancement has enabled the embedding of computing capabilities to existing devices and machines. One of the sectors in which we have experienced great technological progress during the last decades is the automotive industry. A high number (30–50) of embedded computer systems are installed in most cars [1] currently produced, partly to regulate basic features like emissions and safety, but also to provide comfort and convenience to passengers. Vehicles are being gradually equipped with more computing infrastructure to those ends, and the long-term goal is to soon be able to offer autonomous vehicles as a mainstream option for consumers. Such autonomous vehicles will boast twice as many embedded computing systems that will collect and process data at immense rates in order to be able to operate safely.

The currently existing computing capabilities make it easy for vehicles to record data regarding their position and velocity. Such technology is already widely implemented in the US, where every vehicle manufactured since 2014 is outfitted with an event data recorder (EDR), or “black box”, that records a wide range of data to be used (if necessary) in court [2]. Additionally, in many EU countries, insurance companies use such devices to help them design insurance premiums for young drivers [3]. However, the data collected by black boxes are, by default, available to insurance companies regardless of whether or not this is truly required (e.g., an incident has recently occurred).

Given that the technology that senses data is already embedded in vehicles, and the initial attempts to transmit such data to remote locations have been recorded, the next step is to conduct data monitoring in an efficient, global, secure, and privacy-preserving way. Constructing a device that monitors the data provided by sensors embedded in vehicles, encrypts those appropriately, and sends the produced records to a remote secure storage resource is a feasible target. The main requirements would be internet connectivity and sufficient processing resources to perform the encryption of the data.

Recent findings point to a significant rise in the urban population by 2030, which will trigger an increase in vehicular incidents occurring in urban areas. This has motivated researchers to focus on the development of a variety of technologies pertaining to automatic incident detection and management [4]. Such incidents already account for a massive amount of deaths and injuries across the globe. This fact is clearly reflected in the “Global status report on road safety” conducted in 2018 by the World Health Organisation [5]. In the same report, it is stressed that the necessary reform shall be greatly based on the enforcement of safe road user behaviours, which can be increased if liability cannot be avoided. The most pertinent factor in need of monitoring is a vehicle’s speed, which has been proven to directly correlate to the risk of an incident occurring. It is essential that drivers are motivated to adhere to speed limits, and methods like speed cameras have long been employed to that end. There is a common belief that technological measures can greatly contribute toward safer driving behaviours.

Blockchain is a distributed ledger technology that enables the immutable storing of information on cryptographically linked blocks. All records are stored on the network’s peer nodes, making modification of information and identity impersonation impossible. It enables interaction on a zero-trust ecosystem, which lacks a central authority, while also providing resilience to single-point-of-failure scenarios. Two main blockchain approaches exist [6], public (Bitcoin [7], Ethereum [8]), and permissioned (Hyperledger [9], Corda [10]). These approaches are distinguished from each other mainly with respect to who is allowed to participate in and maintain the network. The first poses no restrictions, while the latter operates on an invite-only policy in an attempt to enhance privacy. Blockchain technology’s features such as security, integrity, and auditability set it as a very well-suited platform for applications that handle critical information and involve liability assignment.

The combination of the aforementioned technologies, powerful embedded systems, and blockchains, could have multidimensional benefits. When an incident (such as a car crash) takes place, authorities would no longer have to rely on drivers or descriptions of passersby estimations to conclude which party should be blamed for the incident. At the same time, drivers would feel comfortable and in control regarding the use of their personal information and also be sure that a fair settlement would be more easily achieved by authorities.

In this paper, we present a blockchain-based forensics framework for vehicular related incidents in a smart city environment. The main concept of the framework is to continuously collect vehicle data and store it in an encrypted form on a distributed system that ensures data integrity. Whenever the need for access to the data arises, authorities can request the encryption key from the driver, or retrieve it through a secret sharing scheme, given that a set of organisations (e.g., police, jurisdiction, municipality) agree. The framework also offers an automated way (through smart contracts) to document the incident and liability workflow by the drivers or by assigning liability by the police. The framework is designed upon the Hyperledger fabric platform [9] that offers the required scalability and privacy guarantees. We have also conducted a detailed simulation to assess if such a system would be applicable to an average city and what the resource requirements would be. The traffic data of a full day from the city of Cologne, Germany, have been used to assess the number of transactions and bandwidth requirements that the proposed system would induce for all vehicles in the city.

The main contributions of the present paper are:

- A technically mature approach for decentralized storage and retrieval of vehicles’ data.
- A system that focuses on utilizing gathered data for incident cataloguing, reliable resolving and liability attribution regarding violations involving one or multiple parties.
- The incorporation of a privacy protection layer that enables the system’s adoption with minimal privacy loss for vehicle owners.
- A formal security analysis of the proposed protocol.
- An estimation of the load that such a system would have to cope with if applied in a real world urban environment.

The rest of the paper is structured as follows. Section 2 presents similar efforts in the literature and discusses their offerings; Section 3 gives some background on the core components of the implementation; Section 4 analyses the design of the framework; Section 5 presents the initial implementation steps; Section 6 presents a formal security analysis of the system; Section 7 demonstrates the results from experiments conducted to evaluate the design, and finally Section 8 presents our conclusions and future work plans.

## 2. Related Work

Various similar approaches exist in the literature that either focus on vehicular forensics or aim at providing such functionality for the general smart city ecosystem.

### 2.1. Vehicular Forensics

The authors of [11] propose a framework for determining liability in the event of an accident where an autonomous vehicle is involved. This blockchain-based scheme uses a new consensus mechanism called Proof of Event with Dynamic Federation. The framework forms networks that include, besides the vehicles that participated in the incident, all vehicles that share the same Cellular Base Station with them named as “witnesses”. It makes use of the variety of sensor devices of all said vehicles that belong in the network. The event data generated and broadcasted by them will be included in a new block after verification, which is the responsibility of “verifier” vehicles, which are selected based on a reputation score.

Block4Forensic, introduced in [12], is a framework that aims to facilitate the collection and use of vehicular diagnostic data for resolving incidents. All involved parties, such as vehicles and their manufacturers, insurance companies, maintenance service providers, and law enforcement are brought together in a trust-less environment. A lightweight blockchain is exploited to store cryptographic hashes of car diagnostics as well as maintenance records. Owners of such data can selectively release it to determine the party at fault. However, this framework is not protected against malicious participants and has no means of detecting deviant behaviour.

The authors of [13] furthered their research, and present B-FICA [14], a vehicular forensic framework, based on a permissioned, partitioned blockchain. Various advantages are provided, such as allowing participating parties to share their information only when necessary, guaranteed evidence integrity (thanks to a lightweight consensus and validation protocol), and inconsequential overhead delays. The greatest novelty of the design is that both sensor data and entity interactions are tracked and taken into account during the resolving process. Participants log safety events and evidence, and provide instructions and record their execution. Auto manufacturers and insurance companies use the system to review the data gathered and assign liability. Government transport and legal authorities have the role of validators of the transactions.

The authors in [15] introduce the concept of IoV forensics and present an architecture that audits interactions between sensors, smart vehicles, other smart devices, and the cloud to facilitate evidence handling. The main components in their design are various actors and their interactions. Said interactions are grouped by vehicular event type, combined into “stories”, and eventually stored on a blockchain block. The blockchain itself is hosted in a publishable proof database and guarantees the interactions’ integrity. Proofs for recent blocks are occasionally published on the Internet by the Proof Publisher that maintains the blockchain. Those proofs are stored and utilised by Law Enforcement agencies.

### 2.2. Smart City Forensics

The authors of [15] have extended their work and presented [16], an effort towards a more generic forensics framework. Consequently, they expanded their research [17] by presenting a more advanced design named FIF-IoT. FIF-IoT is a blockchain-based forensic investigation framework that ensures the collection of evidence from IoT devices and offers secure, tamper-proof, and transparent storage procedures. Possible interactions are categorised (IoT device to user/cloud/other IoT device) and logged as interaction transactions containing the action as well as the identities of involved parties, while data stays in an encrypted format. These transactions are periodically collected, compiled, verified, and stored by miners on the blockchain. In the event of a crime or dispute, an investigator can gain access to the decrypted identities and information in a transaction to resolve the matter. The authors provide extensive security analysis and experimental results of a prototype implementation.

The forensic chain presented in [18] aims at tracking digital forensics along the chain of custody. Blockchain is the main technology the framework relies upon to ensure complete transparency when it comes to evidence handling, as every transfer and access attempt for data is recorded on the chain permanently, in an automated manner, thanks to Ethereum smart contracts. Additionally, a hash of the protected evidence is stored on the blockchain, making it easy to verify its integrity.

The BIFF framework, introduced in [19], provides automated evidence gathering from IoT devices. This permissioned blockchain system, which uses the byzantine fault tolerance consensus, focuses on recording the entirety of events in the digital evidence life cycle. Additionally, in an effort to increase privacy, Merkle tree signatures are employed. However, it is not quite a decentralized scheme since there is a Law Enforcement Agency that is in full control of the gathered data.

### 2.3. Review

The majority of existing works related to vehicular/digital forensics fail to extensively predict and guard against malicious scenarios. Most frameworks are not secured against malevolent internal activity, and some are entirely lacking an approach devoted to handling unwanted behaviour/incidents.

Few of the papers discussed have tested the proposed approaches to present a detailed analysis of the performance yielded. Most efforts lack performance evaluating metrics that are very significant for the successful application of the presented schemes. The capacity of such systems, with respect to the number of connected devices, data submission rate, and blockchain infrastructure, should be studied, as it is common for blockchain-based systems to be inefficient, costly, or even inapplicable in a real-world setting.

The majority of authors of the aforementioned works have opted for creating custom blockchain systems instead of using an existing, established one. Such an approach usually facilitates implementation, since it minimizes the requirement of adjusting the proposed system to the capabilities of an existing blockchain network, and eliminates any scalability problems or potentially high costs. On the other hand, it partially sacrifices security and stability, features offered by established blockchain platforms, which are of utmost importance, especially when it comes to handling data that are used for law enforcement purposes.

The present paper proposes a forensics framework for vehicular related incidents. It provides a fair, fast, and automated way to resolve disputes between owners of vehicles and/or insurance companies. The proposed system is based upon existing mature technologies and provides security and trust to participating actors. To the extent of our knowledge, it is the first research proposal in the literature that attempts to:

- **Assess the actual load** (in terms of transactions and network traffic) that such an approach would have. The case of the real-world application of the proposed system for a medium-sized city has been extensively studied to simulate the operation of the system and conclude on the infrastructure requirements.
- **Provide a formal security analysis** of the proposed system. The security properties of the protocol are analyzed, by using an established formal security analysis approach. Existing similar research efforts either completely neglect to take into account required security properties or insufficiently discuss them.

## 3. Background

### 3.1. Hyperledger Fabric

Hyperledger fabric [9,20] is one of the Hyperledger projects hosted by the Linux Foundation. It was initially developed in 2015 by IBM and Digital Asset and it has been mainly designed for enterprise use. Similar to other blockchains, fabric uses a ledger, runs smart contracts, and provides transaction management. However, unlike the firstly proposed blockchain networks, it is private and permissioned, offering identification of participants through a Membership Service Provider (MSP). High transaction throughput rate and fast transaction confirmation time are some of fabric’s most appealing features, along with the fact that it is highly configurable.

The ability to form channels is arguably fabric’s most notable characteristic. It allows the formation of subgroups between participating organisations and the creation of private transaction ledgers for those subgroups. Access to such ledgers is controlled by policies attached to the corresponding channels. There is no limitation regarding the number of channels that can be formed in a fabric network.

Fabric smart contracts are known as chaincodes, and the terms are often used interchangeably. Currently, supported programming languages include Go, NodeJS, and Java. Chaincodes are invoked by clients that are not part of the blockchain network, in order to interact with the ledger. The code of the chaincodes defines and enforces the specifications of such interactions.

The fabric ledger is made up of two separate parts. One is the world state, essentially a database that maintains the current state, and the other is the transactions’ log, a blockchain that records all committed transactions that have driven the ledger to its current state. Each channel comes with its own ledger which is distributed to all the participating peers of organisations that have access to it.

Peers (or peer nodes) contribute a great deal in a fabric network. Ledgers and smart contracts are hosted by them, and they act as necessary intermediaries for out-of-network clients that wish to interact with a chaincode. Many different kinds of peers can exist in a network, with the most important ones being committing and endorsing peers. Regardless of its type, every peer belongs to, and is maintained by, an organisation.

Organisations are the main entities that form a collaboration and maintain a shared fabric network. Each organisation provides its MSP for identity management and maintains a number of peers and ordering nodes. MSP is provided through trusted Certificate Authorities (CAs), which are operated by each organisation separately, and issue X.509 compliant certificates for all the identities of the organisation that are going to interact with the network. CAs play an integral role as the aforementioned certificates enable the establishment of access control mechanisms on the network.

In Hyperledger Fabric, a set of ordering nodes is in charge of forming blocks of transactions. Those make up what is called the ordering service of the system. The nodes comprising the service can come from any number of different organisations/members of the network. The ordering of the transactions maintains the sequence in which the transactions arrive. A single ordering service exists per network and handles transactions for all its channels. Blocks generated by the ordering service are indisputable, thanks to the deterministic nature of the algorithms used by fabric for this process.

Finally, private data collections play an important part in Hyperleger fabric as they allow the exclusive and private exchange of information between a subgroup of channel members. The private data is stored by authorised organisations’ peers in a private state database, and all other peers, orderers included, are incapable of viewing it. However, a hash of the private data is publicly stored in everyone’s ledger, to ensure private data integrity.

### 3.2. IPFS

The Interplanetary File System (IPFS) [21] is a peer-to-peer file system protocol that aims at providing a fast, secure and resilient storage resource for all peers. Files are content addressed instead of using a name or location. Thus, it is infeasible to accidentally or maliciously alter data in place, as that action would directly alter the identifier of the file itself. On the other hand, this adds another layer of complexity related to finding the location of a file in the distributed network, which is solved through a routing system that can retrieve other peer network addresses and whether those can serve a specific file. IPFS achieves routing using Distributed Hash Tables based on S/Kademlia [22], and Coral [23], and secure human-readable identifiers through IPNS, a decentralized version of DNS.

In IPFS, data are stored as IPFs objects, which are data blocks that do not exceed 256 KB in size. In order to construct structures for larger files, or even directories, such blocks may be connected through links that enable the connection of multiple blocks together. The objects that are connected together form a Merkle DAG. In IPFS, if one node is down, other nodes in the network can serve files. Thus, data blocks still exist on the network as long as there is at least one node that stores it. IPFS also eliminates content duplication, as files with exactly the same content are stored only once. Distributing the data blocks of a large file to multiple different peers, facilitates the efficient retrieval of the file.

In general, IPFS offers an efficient and secure storage resource of theoretically unlimited size that is characterized by very high availability. The storage nodes operate on a peer-to-peer basis, and no node is able to tamper with the stored data. Because of such characteristics, IPFS is commonly proposed to be used along with blockchain systems (that have storage limitations) and to serve as a storage back-end that will guarantee the integrity of the stored data.

### 3.3. Secret Sharing Schemes

Secret-sharing is a methodology that enables an entity to create a number of shares out of a secret system, a predefined number of which can be used for the reconstruction of the initial secret [24]. The main reason such schemes are used is the requirement of the collaboration of more than one party for the retrieval of secret information. This actually enforces a higher level of security against malicious actors that attempt to reveal secret information.

Such schemes are usually described as t−out−of−n schemes which have the combination of at least *t* out of *n* secret shares required to reconstruct the initial secret. The main properties such a system shall hold are:

- **Correctness:** The secret can be determined by any combination of *t* shares out of the complete set of *n* shares. There exists an algorithm that efficiently computes the secret from the *t* shares.
- **Privacy:** Access to any t−1 shares out of the complete set of *n* shares gives no information about the value of the initial secret.

Secret sharing schemes are mainly used as part of secure protocols, in order to enhance their resilience to secret leakage attacks [25].

## 4. Framework Design

In the present Section a high-level description of the system, the actors, and the various processes that take place are given, while no actual algorithms or technical decisions for the system are discussed. In the next Section all the implementation details for the proposed system are covered, such as the choice and configuration of the blockchain platform and storage network along with the selection of cryptographic algorithms used in the system.

### 4.1. Overview

The present paper introduces a blockchain-based forensics framework for vehicular related incidents in a smart city environment. Incident-related data are recorded in a secure, private way that guarantees integrity and availability. Through fast, immutable, and decentralised procedures, the proposed scheme enables a privacy-preserving approach that can provide valuable information for a vehicular related incident, such as a car crash, when required. It serves as a storage resource for critical information while allowing access to said information only when necessary and solely for entities that have the proper rights to it.

The main concept of the framework is that vehicle movement data are continuously encrypted and stored on a distributed network that ensures data integrity. Whenever a car crash incident happens, access to such data can facilitate the fast and automatic resolving of a dispute between the involved vehicle owners and the corresponding insurance companies. Access to collected data can normally be achieved through the consent of the vehicle owners. In cases where this is not feasible, regulatory authorities can decrypt stored data, given that there is a strong consensus between them. Decryption by a single entity (other than the driver) is not allowed—in order to avoid potential privacy violations. Authorities are able to conduct sample checks by requesting access to past data, to check if vehicles are reporting in a valid way. This can happen through cross-correlating submitted data with data from either other vehicles or other sources (e.g., traffic cameras), but the actual cross-checking is out of the scope of the present paper.

### 4.2. Components

The proposed system is based on three main components, a blockchain network, a distributed file storage system, and an IoT device installed in vehicles.

The blockchain network of the system has been designed and developed using the Hyperledger fabric [9,20] platform. A fabric network is set-up and maintained by the system actors, which participate in it either through maintaining a peer of the system or interacting with it. Smart contracts that orchestrate both data collection and data retrieval are deployed on the fabric network.

Directly storing large volumes of collected data on the blockchain is an inefficient choice, especially for the use case under consideration. Instead, we have opted for the Inter-Planetary File System (IPFS) [21] to be utilized as the main storage resource of the system. Setting up an IPFS network allows the storage of sizable and sensitive data on a distributed file system, that can guarantee both data availability and data integrity. Integration of the storage layer of the system with the blockchain layer enables the secure and efficient processing of large volumes of data.

The last component of the implementation is an IoT device, that is installed into every licensed vehicle in the smart city. This device’s main functionalities are data collection from the vehicle’s sensors (such as its location and velocity), data encryption, and data uploading to the IPFS. The device also renews encryption keys periodically and is responsible for key management and transfer to the IPFS network. Finally, this device ultimately commits the required data to the fabric blockchain. For the present paper, the device has been emulated by a software agent, to design the general framework. A capable hardware implementation of the device will be proposed in our future work.

### 4.3. Actors

The framework is designed to function for the benefit of various different entities, all of which are involved in vehicular related incidents. Each of the actors holds a private/public key pair that is used to selectively allow them to access the publicly available data in the storage component of the system.

#### 4.3.1. Vehicle Owners

All owners of vehicles in the smart city environment have access to the information gathered by their own vehicles’ sensors and can use such data to speed up the process following an incident by sharing them with the authorities. In all cases, they benefit from the fast settlement of incidents that the scheme provides and can depend upon the integrity of the evidence stored in the system.

#### 4.3.2. Insurance Companies

Insurance companies are brought in for the purpose of providing the required infrastructure for the set up of the fabric network. They are given access to data for the incidents their clients are involved in, and can use them to carry out their vehicle insurance procedures. Since the data stored in the system cannot be tampered with, the information can be trusted and used as fair decisions for all parties involved.

#### 4.3.3. Authorities

This category includes an abundance of different agencies such as law enforcement, traffic police, judicial authorities and the municipality, all grouped together and referred to as authorities for the sake of simplicity. Authorities are the closest to an administrative entity, as they have administrative rights over the fabric network. Authorities gain access to vehicle-related data when deemed essential, to handle a case. Additionally, through the use of smart contracts, they generate additional incident-related data (such as involved vehicles, time, etc.) and store those in the system. Authorities also participate in a secret sharing scheme that functions as the last resort procedure that enables key retrieval, required to access critical encrypted data, even if vehicle owners do not consent to that retrieval.

### 4.4. Actors’ Interaction

**Figure 1:**
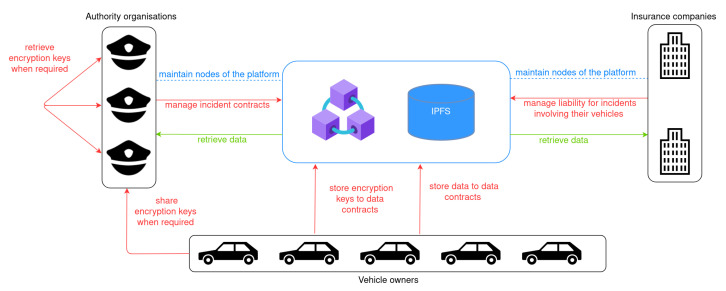
Actors and interactions with chaincodes.

The interactions of each system actor in the platform are depicted in Figure 1. Specifically, the vehicle owners’ main interaction is to store data and encryption keys in the platform and potentially share the encryption keys with authorities when it is required. Authorities manage incident contracts, filling them in with event-related information that they collect upon an incident occurrence. They also collaborate to retrieve encrypted data when vehicle owners are not able or willing to collaborate. Insurance companies interact with incident contracts to assume liability in an incident case.

Authority organisations and insurance companies are also responsible for maintaining the nodes of the distributed platform and thus guarantee the integrity of stored data. Each one of those organisations maintains a node in the blockchain network and a node in the IPFS network, while the insurance companies are responsible for maintaining the data of their vehicles in their IPFS node.

### 4.5. Functionality

The main functionality of the system can be split into three distinct phases, data collection, key management, and data retrieval, which are analyzed in the present Subsection.

#### 4.5.1. Data Collection

**Figure 2:**
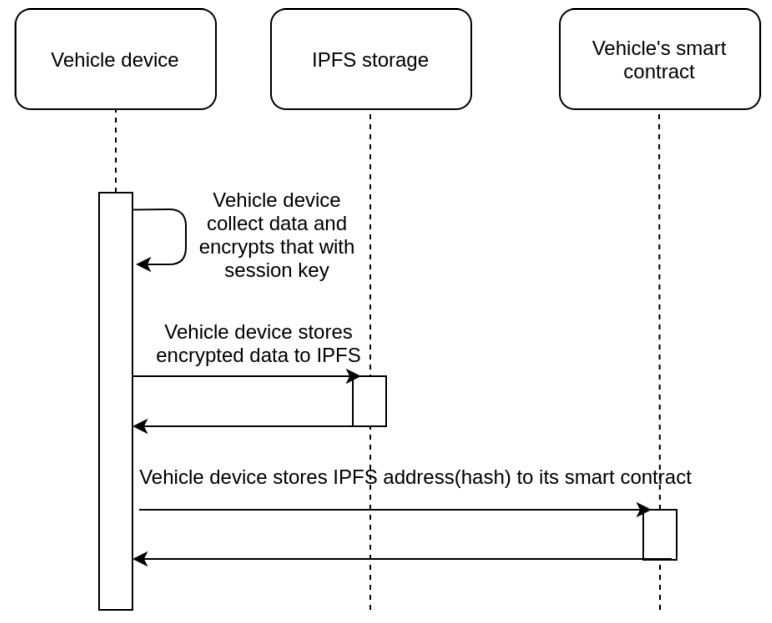
Data collection sequence diagram.

Data collection, through embedded sensors in the vehicle, is the main procedure taking place. The location and velocity of vehicles are constantly recorded by the IoT device of the scheme. The collected data are encoded as a bit array depicted in Equation 1, where $X_{m}$ and $Y_{n}$ stand for the binary representation of longitude and latitude coordinates of the location of the vehicle and $V_{8}$ stands for the binary representation of the velocity of the vehicle. The length of $X_{m}$ and $Y_{n}$ is dependent on the size of the area the system should support, while the length of $V_{8}$ is fixed, as it can support velocity values up to 255 km/h. The aforementioned values are monitored and stored as integers, as further precision is not required. The optimal value of *m* and *n* that can support large smart city areas are around 20 bits, and thus, 48 bits are sufficient for any use case.The operations the device performs using the data are depicted in Figure 2 and are listed below:

(1) The data collected are encrypted to prevent exposure to third parties. Encryption happens through the use of a symmetric session key that is periodically refreshed, as described in the next Subsection.
(2) The encrypted data are stored on the (IPFS) network. A hash that points to the IPFS file where the data can be found is retrieved by the device.
(3) The IPFS hash, along with a timestamp that defines when the data were collected, are stored on the fabric blockchain for future use.

(1) $$X_{m}\left|Y_{n}\right|V_{8}$$

This course of action takes place every second to ensure that relevant data is always accessible when required and no information is lost. Hence, even in the worst-case scenario when an incident takes place and the device is destroyed due to severe damage, data up until the previous second will be available on the system’s storage. For efficiency reasons, we have opted to store a data point to the infrastructure of the system only if the velocity of the vehicle has changed from the previous data point. A vehicle moving with a constant speed enables an accurate estimation of its metrics by the system and thus the corresponding data submission is omitted and the data point is replaced by its estimation if it needs to be retrieved. This approach enables a more efficient operation of the system, with minimal accuracy deviation.

#### 4.5.2. Key Management

**Figure 3:**
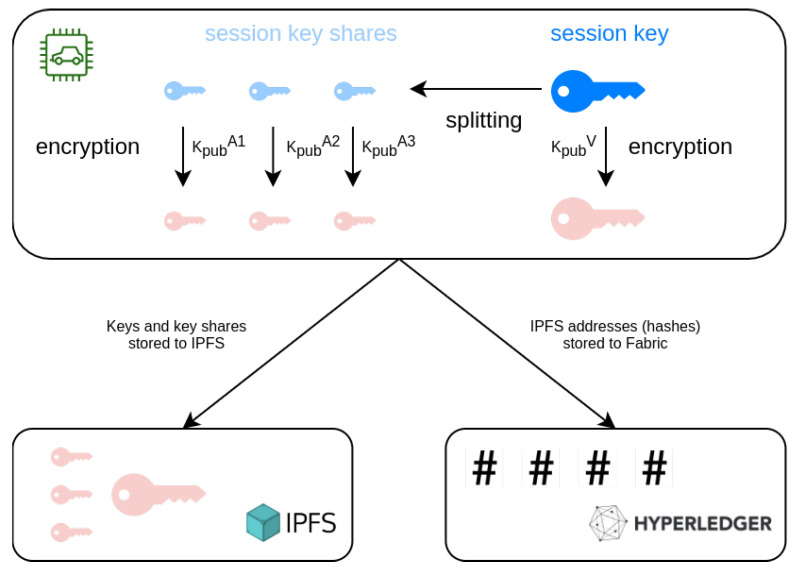
Session key management.

In order to protect the users’ privacy, the vehicle device produces a new session key $K_{s}$ every hour. Session keys are generated on the vehicle device and then used to encrypt collected data of the time window during which they are valid (e.g., for the next hour). Therefore, even when an entity is given access to view the information it is entitled to, this can be done for only a limited time frame. The session key handling is depicted in Figure 3.

For every $K_{s}$ generated, a series of operations is enforced. Firstly, the new key is encrypted with the vehicle owner’s public key $K_{pub}^{vo}$, thus owners maintain an unrestricted view of their personal data. Additionally, they maintain the option to reveal any time-frame’s key to authorities, granting access to said data in an automated and fast way.

Furthermore, to enable an emergency alternative for cases when a vehicle’s owner is disinclined or unable to provide the keys to authorities, we apply a secret sharing scheme. Secret sharing is the method of dividing a secret between various participants while making sure each individual share cannot be used to retrieve any information about the secret, but that a combination of a predefined number of shares can reveal it. In this case, participants will be high-level authorities (e.g., judicial authorities, law enforcement, traffic police) and the secret is the session key. When the device produces a new session key it creates shares and each of the individual shares is encrypted with the corresponding authority’s public key $K_{pub}^{a}$.

Similar to the vehicle-related data, encrypted keys and encrypted key shares are stored on the IPFS. The corresponding hashes are stored on the blockchain, along with a timestamp defining the moment the keys became active, to facilitate retrieval of a specific time slot’s session key for a vehicle.

If circumstances demand it, a predefined majority of authorities can collaborate, retrieve, and decrypt a number of shares that can enable the session key reconstruction. For the purposes of the paper, we opted for requiring more than half the authorities of the ecosystem to collaborate through share joining, in order to retrieve a session key.

#### 4.5.3. Data Retrieval

The process of retrieving the encrypted data from the system is a matter of gaining access to the proper encryption keys, as access to stored data is granted for all. The ability to get access to the active or past session keys will be required by the aforementioned authorities for two purposes:

- **Incident:** In the case of an incident, it is necessary to gain access to the information stored by the involved vehicles’ devices, in order to facilitate the process of determining the liable party/ies.
- **Validity control:** As it is possible for vehicle devices to not operate in a proper way, either due to malfunction or being tampered with by the vehicle owner, a control process is required to check the validity of the data being submitted or to detect the deactivation of the device (the absence of data). The responsible authority (e.g., the traffic police) may ask vehicle owners to retrieve, decrypt, and send past session keys, for checking the validity of the submitted data. Detecting inconsistencies that come up by observing approximate vehicle data, or cross-correlating reported data with other sources (e.g., traffic cameras, manual observation) will enable the identification of malfunctioning vehicle devices. The methodology of validity control is outside the scope of the present paper.

There are two different procedures through which an authority can gain access to encrypted data for a specific vehicle, depending on whether the vehicle owner provides their consent.

**Figure 4:**
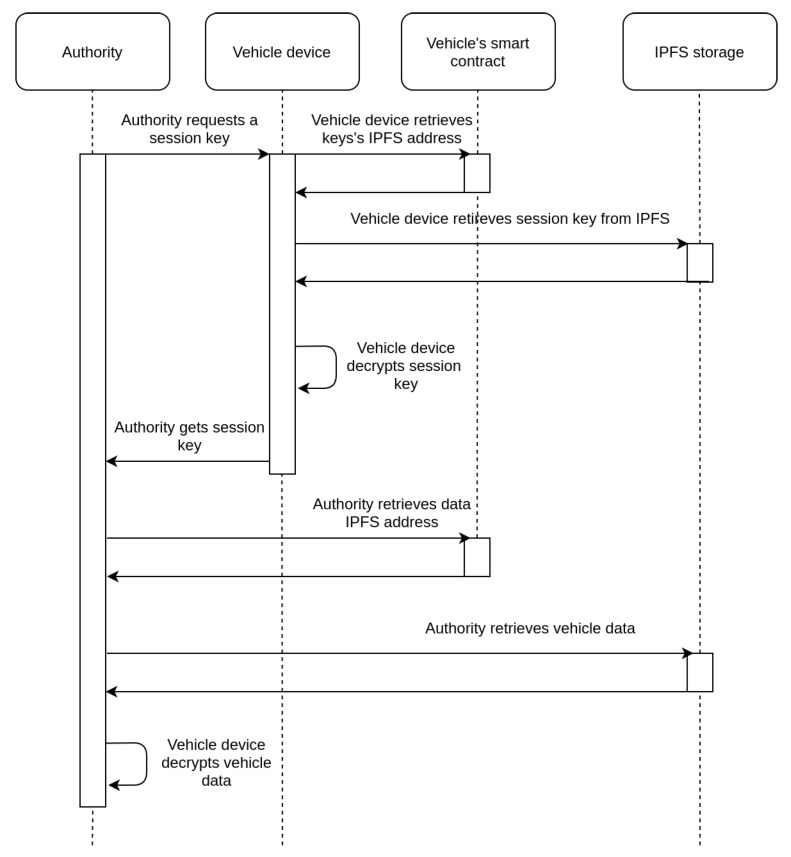
Sequence diagram for retrieval of data given the consent of the vehicle owner.

**Figure 5:**
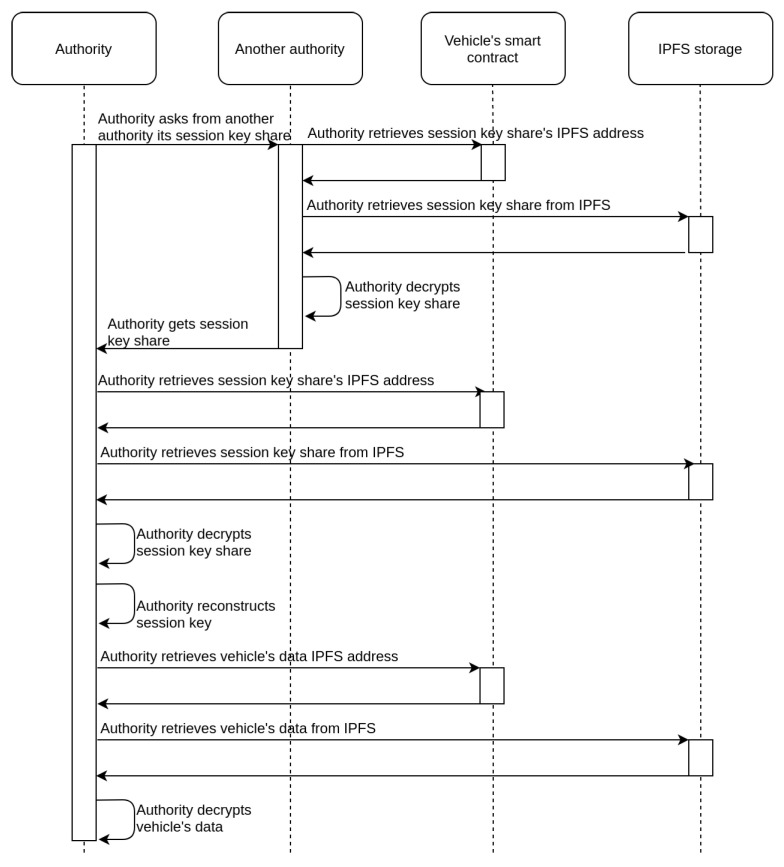
Sequence diagram for retrieval of data without the consent of the vehicle owner.

- The first option is to get access through the consent of the user and the corresponding process is depicted in the sequence diagram of Figure 4. Owners of vehicles can retrieve information collected by their vehicles at any given time, as each session key $K_{s}$ used, has been encrypted with the user’s public key and stored on the IPFS system. When the vehicle owner is requested to provide access to an authority (regarding data collected at a specific time point), their vehicle’s device retrieves the IPFS address of the corresponding $K_{s}$ from the smart contract, downloads the encrypted $K_{s}$ from the IPFS system, decrypts it with a private key $K_{pri}^{vo}$, and submits it to the authority. The authority is then able to access the data collected by the specific vehicle for the corresponding time slot.
- In cases where the vehicle owner is not willing or able to provide the requested $K_{s}$ to the authority, there is an alternative approach, which is depicted in the sequence diagram of Figure 5. This takes advantage of the secret shares of the $K_{s}$ stored on IPFS. If an authority (e.g., police) can prove to other authorities that there is a need for accessing a vehicle’s data, then it is feasible for them to collaborate to achieve it. Specifically, each authority that approves the request for data access can retrieve its share from the IPFS, decrypt it, and provide the requesting authority with the result. Given that a sufficient number of authorities approve the request, the $K_{s}$ can be reconstructed and the collected data can be retrieved. In Figure 5, for simplicity reasons, there is an assumption that only two shares are required to reconstruct the secret, so the authority needs to contact only one additional authority.

The default procedure to retrieve the vehicle data is the first one. Given the consent of the user, the authority organisation can retrieve the required data in most cases. Only in extreme cases, where the user is not willing/able to collaborate with the authority organisation, is the second procedure activated. This procedure has an increased communication overhead and also creates privacy violation implications, and thus, cannot be used as the default option. The rest of the organisations agree to collaborate by sharing their secret shares only when accessing the vehicle’s data is fully justified and the first procedure is impossible.

#### 4.5.4. Incident Handling

In case of the occurrence of an incident, it is necessary to have all the pertinent details documented. This is achieved through the use of the Incident contract.

An incident contract is instantiated by the authority on the site of the incident. Since such a contract will not be frequently interacted with, the load it will induce to the hosting blockchain network chain is not significant. Nevertheless, it is important that this contract can be easily located when needed, hence it is suggested that it is stored in a separate fabric installation solely dedicated to the purpose of maintaining incident contracts, but any scheme preferable to the system’s users is feasible.

Following instantiation, the incident contract needs to be filled in. This is also performed by authorities that collect the necessary keys, either from the involved parties willingly share them, or receive through the secret sharing scheme. The information is then retrieved from the data contracts and transferred onto the new incident contract.

This process may initially seem like it is placing trust on the authorities responsible for the logging of data on the incident chaincodes. However, this is an inaccurate assumption. The immutability of the blockchain ledger guarantees that any information on the incident contract can always be cross-referenced with the original entry on the data contract that is unaltered.

In addition to the information related to the incident, an incident contract also holds the outcome or liability distribution amongst involved parties. Any one of them has the option of assuming complete or partial responsibility directly in the contract through the use of their private keys, or liability, can be assigned at a later time by the relevant authority or insurance company.

An instantiated and filled-in incident contract serves as a reference point for a vehicular incident occurring in the monitored area, gathering all relevant input and outcomes in a single immutable and accessible storage point.

## 5. System Implementation

The present Section discusses the implementation of the proposed system. The main component of the framework is a Hyperledger fabric blockchain network, which is integrated with an IPFS distributed file storage network. On top of that, encryption and secret sharing schemes are deployed, to facilitate the implementation of an access control mechanism for the data produced by the vehicles’ devices.

### 5.1. Structure of the Fabric Network

**Figure 6:**
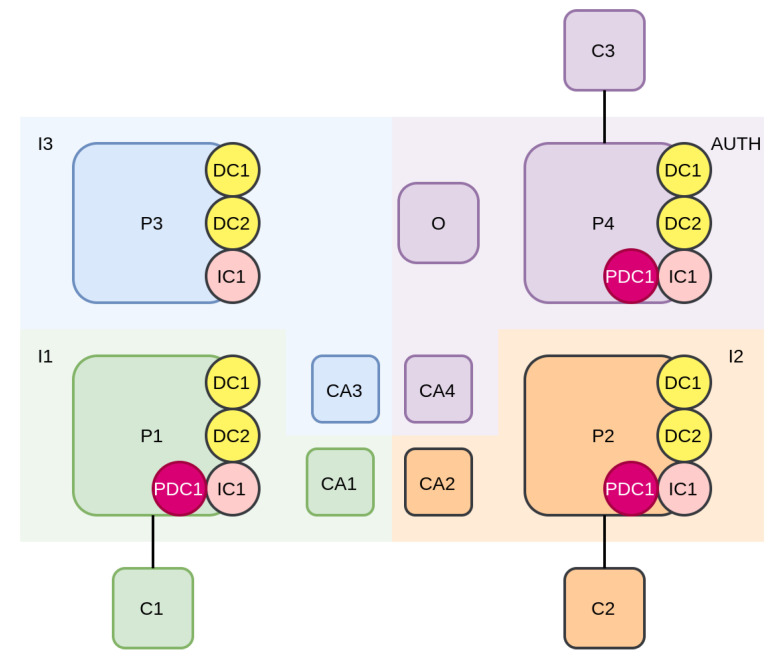
Layout of Hyperledger fabric network.

The structure of the Hyperledger fabric network is depicted in Figure 6. A fabric network is maintained by a group of collaborating organisations. For the proposed system the main organisations that operate the fabric network of the proposed system are insurance companies. They take advantage of the system, to automate their interactions and interact in a fairer and more secure way. Besides the insurance companies, at least one trusted regulatory organisation, that holds a central role in the system, is required. This entity can, for example, be the police or municipality. In order to fully take advantage of the system’s offerings, such as data retrieval without the vehicle owner’s consent, the participation of more than one regulatory organisation is required.

In Figure 6, an example setup with four different organisations is depicted. They are three distinct insurance companies I1 (green), I2 (orange), I3 (blue), and a regulatory authority AUTH (purple). The insurance companies host one peer node each (P1, P2, and P3, respectively) and one CA node each (C1, C2, and C3, respectively). The regulatory authority, apart from maintaining its own peer (P4) and its own CA (CA4), takes up specific tasks in the network, such as hosting the Ordering Service (O), and administering the fabric network’s configuration.

As discussed, every participating organisation maintains its own Certificate Authority, colored according to the organisation it belongs to. CAs serve the network by issuing certificates for all identities connected to their organisation. Insurance companies manage the identities of their personnel but are also responsible for the identities of vehicle owners that are contracted to them. AUTH organisation’s CA (CA4) only manages the identities of the authority organisation personnel.

The main actors/systems that interact with the platform, are the devices of the vehicles that are contracted to the aforementioned insurance companies and the personnel of both the insurance companies and the regulatory authority. For each vehicle, there is a vehicle device (client) that senses data during vehicle operation and submits those to the chain. In Figure 6, two such client applications are depicted, C1 and C2 that belong to I1 and I2 (their colors correspond to the organisation each one belongs to). C3 represents the client of the personnel of the authority organisation AUTH.

During the initial deployment of the network, as seen in Figure 6, a single channel is set-up and shared among all participating organisations. The administrative rights for this channel are assigned to the authority organisation (AUTH). This means that only the AUTH organisation can add new members/organisations (new insurance companies) to this channel. All members of the network become members of the channel, and install in their peers the smart contracts they are concerned with and have been allowed access to. Additionally, all peers belonging to the channel host the channel’s ledger. The smart contracts deployed in this single channel can be divided into two categories: Data Contracts and Incident Contracts.

#### 5.1.1. Data Contracts

For each vehicle, there is a smart contract deployed to the single channel. An IoT device equipped in each vehicle monitors data, encrypts data, and stores the corresponding file to the IPFS network. In order to facilitate the retrieval of such data, the hash (IPFS address) of each data file, and the corresponding timestamp, need to be stored in a secure way; data contracts are the means to that end. For each vehicle that participates in the framework, a new data contract is instantiated on the common channel and installed on all peers.

In Figure 6, four peers (three of the insurance companies and one of the authority organisation) are depicted. Data collection smart contracts (DC1,DC2) correspond to two different vehicles (C1 and C2), which are contracted to I1 and I2. Those smart contracts are stored in every peer, irrespective of the insurance company the vehicles are contracted to.

The functions of the smart contract that enable the aforementioned functionality are the following:

- **storeData**: This function accepts the timestamp and IPFS address of the encrypted data as input and stores them to a mapping in the contract. The function can only be called by the vehicle device the contract belongs to.
- **storeKeys**: This function accepts the timestamp, IPFS address of the encrypted session key, and a set of pairs of authority organisations ids and IPFS addresses of the corresponding session key shares as input. It is called in one-hour intervals to store the newly generated session key information and is only accessible by the identity of the device of the vehicle the contract belongs to.
- **retrieveData**: This function is used to retrieve the IPFS address of the vehicle-related data for a specific timestamp.
- **retrieveSessionKey**: This function is used to retrieve the IPFS address of the files containing the session key and session keys shares for a specific time interval.

#### 5.1.2. Incident Contracts

For each vehicular incident, a new smart contract (Incident contract) needs to be instantiated to the channel. Incident contracts serve the purpose of managing the collection, storage, and viewing of incident-related information. The responsible authority organisation (e.g., traffic police) sets up the new incident contract in case of an event and stores in it the details of the case.

Apart from the typical case data, incident contracts include a private data collection that is only accessible by the organisations of the two insurance companies the two involved vehicles are contracted to and for the authority organisation. This private data collection is destined to store the session keys $K_{s}$ under which the incident-related data have been encrypted for the involved vehicles.

As depicted in Figure 6, if an incident happens between two vehicles (C1 and C2), a new incident smart contract IC1 is deployed by the authorities organisation. This smart contract is deployed to all peers of all organisations. In order to protect the privacy of involved vehicle owners, the incident contracts contain a private data collection. For IC1, this private data collection is noted as PDC1 and is accessible by only I1, I2 (the insurance companies of the involved vehicles), and AUTH (the authority organisation).

The data structure for PDC1 is only stored in the peers of the involved organisations I1, I2, and AUTH, to prevent access to data of the incident for organisations that are not related to the incident (e.g., I3). PDC1 is not present in the peer P3. The use of private data collection enables access control on a per organisation basis (e.g., I3 does not have access to the data of the incident smart contract deployed for the event between C1 and C2). Access control on a per identity basis (members of organisation) is achieved through fine tuning the implementation of the functions of the smart contract (e.g., permitting only C1, C2, and C3 to have access to the data and not allowing any other identity, such as a device of another vehicle of I1 to access the incident’s data).

An incident smart contract has a two-folded use; it serves as the storage of the incident data and also enables interacting parties to settle with regards to the liability pertaining to the incident. The data stored in the contract are the actual session keys for the two vehicles, that were active during the incident. The two session keys enable participants to decrypt the actual monitored data of the two vehicles. The incident smart contract also enables the two vehicle owners (or the insurance companies on their behalf) to assume liability for the incident on a 0–100 scale. Given that the two parties assume liabilities that sum up to 100, the incident is marked as resolved. In the case where this is not possible, the authority organisation can assign the liability scores to the two parties, without their consent.

A short overview of the functions implemented in the Incident Contracts:

- **assumeLiability**: Enables one of the involved parties (or their insurance company on their behalf) to assume liability, thus facilitating the resolution of the incident. This can only be called by the vehicle owners’ identities or the corresponding insurance companies’ personnel.
- **assignLiability**: Allows the authorities (e.g., traffic police) to disperse liability amongst the vehicle owners, in the case the latter cannot reach a consensus by themselves. This function can be called by the traffic police personnel.
- **shareKey**: Parties (vehicle owners or authorities), that have access to the session keys $K_{s}$ under which the incident-related data have been encrypted, use this function to store the keys in the Private Data Collection. This function can be called by either the vehicle owners or the authorities’ personnel.
- **retrieveKey**: Allows participants to retrieve the session keys of the two vehicles. This is only accessible by the vehicle owners or the authorities’ personnel.

### 5.2. IPFS Network

An important component of the network is the IPFS storage infrastructure. Each insurance company takes part in the formation of the IPFS network by providing at least one node. The authorities organisation that administers the fabric network has a central role in the IPFS network as well, as it maintains one or more bootstrapping nodes. These nodes publish a bootstrap list of peers that enables the IPFS daemon to learn about other peers on the network.

The nodes of each insurance company are obliged to pin (keep stored) the information submitted by the vehicles (monitored data, session keys, and key shares) that are contracted to the company for a predefined time period. Failing to properly retrieve such data during data retrieval triggers penalties for the corresponding insurance company. The integrity of the submitted data is secured as the address of each file in IPFS equals the hash of the file securely stored in the smart contract of each vehicle during monitoring. The insurance company, or any other participant, cannot tamper with the content of the files, as such an event would be easily detected.

**Figure 7:**
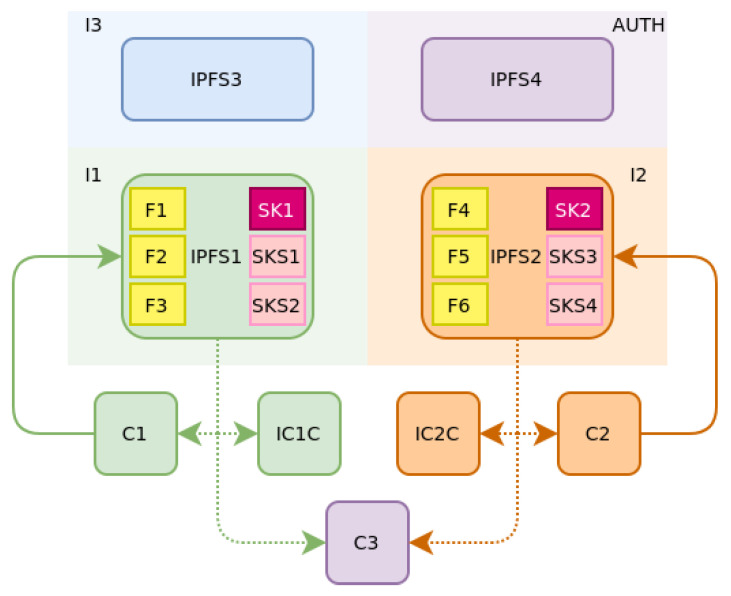
IPFS network structure.

A sample architecture of the IPFS network is presented in Figure 7. Each one of the aforementioned organisations I1, I2, I3, and AUTH maintain an IPFS node which are denoted as IPFS1, IPFS2, IPFS3, and IPFS4, respectively. Node IPFS1 is obliged to hold all files submitted by C1 (vehicle contracted to I1). Assuming that there are more than one authority organisations, the files are data files (F1, F2, F3) containing C1 vehicle’s monitored data, session key files (SK1) for vehicle C1 and session key shares files (SKS1, SKS2) for vehicle C1. Respectively, node IPFS2 holds the files submitted by client C2. In the case of an incident that will involve C1 and C2, the files mentioned above are (potentially) retrieved by various clients.

First of all, C1 and C2 probably require retrieval of the submitted data. On top of that, C3, on behalf of the AUTH organisation and clients (noted as IC1C and IC2C, on the side of the two insurance companies I1 and I2, respectively) are also going to retrieve the data. The flow of data between the client and the IPFS nodes is also depicted in Figure 7. Solid lines show the continuous reporting of data, while dotted lines show the potentially required retrieval of data in the event of an incident.

### 5.3. Cryptographic Algorithms

As discussed in previous sections, the proposed system requires three cryptographic algorithms to be set-up, in order to protect the privacy of vehicle owners. Those algorithms are:

- A symmetric encryption algorithm for the encryption of the submitted data;
- A public key encryption algorithm for managing data exchange between participants;
- A secret sharing scheme to enable the retrieval of data, without the driver’s intervention, if it is required.

With regards to the symmetric encryption of data, there is a strong requirement for fast performance. This is because the encryption operation has to be conducted in every time slot of the monitoring process (which has been set to one second). Out of all symmetric encryption algorithms, AES has been proved to offer the best combination of security and performance in the restricted hardware environments [26,27]. Because of this, we have opted for using the AES-256 cipher for encrypting the data monitored by vehicle devices.

The public key encryption algorithm is used by the devices only when a new session key is generated. During session key generation, n+1 encryption operations are required, where *n* is the number of authorities, as one session key share is encrypted for each authority aside from the session key generated. This happens once every hour, so there is no significant overhead. The only consideration that has to be taken into account is that the chosen algorithm shall be capable of encrypting the session key and the corresponding session key shares, which are 256 bits long. The algorithm chosen was RSA-4096, as it is capable of encrypting the required information, while also offering a great level of security.

Finally, with regards to the secret sharing scheme, we opted for the Shamir Secret Sharing Scheme [28], which enables the split of session keys in multiple different shares. Again, this is a process that takes place only when refreshing the session key, once per hour, so the performance is not critical.

## 6. Security Analysis

In this Section, a security analysis of the proposed framework is conducted, to prove that it protects all interacting parties from the malicious behaviour of their counterparts.

In order to prove the validity of the system from a security point of view, we have opted for describing the procedures of the proposed protocol through the High Level Protocol Specification Language (HLPSL) [29], which is an expressive language for modeling communication and security protocols. Then, with the use of the symbolic model-checker OFMC [30], we have proved that the protocols successfully abide by security requirements such as secrecy of information or authenticity of users/information. Because HLSPL notation is rather complex, we have opted for presenting the protocols in CAS+ format [31], which resembles an Alice-Bob notation and is easier to understand. The AVISPA tool used converts protocols between the two formats.

### 6.1. Threat Model

We assume that vehicle owners, insurance companies, or authorities may become malicious and attempt to either tamper with data to deny liability or violate the privacy of others. On top of that, we assume that it is possible for actors to collude, in order to tamper with or access data they should not.

Specifically, a vehicle owner may attempt to lie about their vehicle data, deny revealing their session key, or tamper with the on-vehicle device to report false data. Insurance companies may not follow up with previous consent about responsibility for an accident, attempt to violate users’ private location data, or attempt to delete files to avoid covering liability costs. An authority may attempt to violate users’ private location data, with no proper reasoning. Additionally, on top of the aforementioned cases, we also assume a couple of collusion scenarios:

- A vehicle owner colludes with their insurance company to escape liability by altering IPFS files;
- A vehicle owner colludes with authorities to escape liability;
- Authorities collude with insurance companies to frame a vehicle owner.

### 6.2. Analysis

The proposed protocol has been expressed in CAS+ as three distinct processes: Listing 1 depicts the data collection process, Listing 2 depicts the data retrieval with vehicle owners consent, and Listing 3 depicts the data retrieval without the vehicle owners consent. In the protocols, an assumption that three authorities exist has been used. The roles used are VO for the vehicle owner, B for the blockchain system, and A for one authority (or A1, A2, A3 for a set of three authorities, according to the scenario). Data variable stands for the data record of the vehicle, and Ks is the symmetric session key used, while Ks1, Ks2, Ks3 are the session key shares produced for the authorities. Finally, Kp is the public key of the vehicle owner and Kp1, Kp2, Kp3 are the public keys of the three authorities.

Given the fact that data integrity is ensured by the blockchain network, the OFMC symbolic model-checker tests the privacy requirements for the aforementioned processes. It is required that access to data submitted by vehicles is not permitted for the intruder at any step of the three workflows.

**Listing 1.** CAS+ definition for data collection processes.
protocol data_collection;
 
identifiers

 VO, B, A1, A2, A3  : user;

 Data          : number;

 Ks           : symmetric_key;

 Ks1,Ks2,Ks3       : number;

 Kp,Kp1,Kp2,Kp3     : public_key;
 
messages

 1. VO -> B  : {Ks}Kp

 2. VO -> B  : {Ks1}Kp1

 3. VO -> B  : {Ks2}Kp2

 4. VO -> B  : {Ks3}Kp3
 
knowledge

 VO   : Data,Ks,Ks1,Ks2,Ks3,Kp,Kp1,Kp2,Kp3;
 
session_instances

 [VO:vo,B:b,A1:a1,A2:a2,Data:d,Ks:ks,Ks1:ks1,Ks2:ks2,Ks3:ks3,\\

 Kp:kp,Kp1:kp1,Kp2:kp2,Kp3:kp3];
 
intruder_knowledge

 vo,b,kp,kp1,kp2,kp3;
 
goals

 secrecy_of Data


**Listing 2.** CAS+ definition for data retrieval process (with consent).
protocol data_retrieval_consent;
 
identifiers

 VO, B, A  : user;

 Data     : number;

 Ks      : symmetric_key;

 Kp      : public_key;
 
messages

 1. B -> VO   : {Ks}Kp

 2. B -> VO   : {Data}Ks

 3. VO-> A    : Data

knowledge

 B  : Data,Kp,Ks;

session_instances

 [VO:vo,B:b,A:a,Data:d,Ks:ks,Kp:kp];
 
intruder_knowledge

 vo,b,kp;
 
goals

 secrecy_of Data


**Listing 3.** CAS+ definition for data retrieval process (without consent).
protocol data_retrieval_no_consent;
 
identifiers

 VO, B, A1, A2, A3  : user;

 Data      : number;

 Ks      : symmetric_key;

 Ks1,Ks2,Ks3   : number;

 Kp,Kp1,Kp2,Kp3   : public_key;
 
messages

 1. B  -> A1  : {Ks1}Kp1

 2. B  -> A2  : {Ks2}Kp2

 3. B  -> A3  : {Ks3}Kp3

 4. A2 -> A1  : {Ks2}Kp1

 5. A3 -> A1  : {Ks3}Kp1

 6. B -> A1   : {Data}Ks
 
knowledge

 B   : Data,Kp1,Kp2,Kp3,Ks;
 
session_instances

 [VO:vo,B:b,A1:a1,A2:a2,A3:a3,Data:d,Ks:ks,\\

 Kp:kp,Kp1:kp1,Kp2:kp2,Kp3:kp3];
 
intruder_knowledge

 vo,b,a1,a2,a3,kp,kp1,kp2,kp3;
 
goals

 secrecy_of Data


## 7. Experiments

It is obvious that a system like the one proposed will suffer under high workloads. Theoretically, in a smart city environment, thousands of vehicles move around concurrently, and thus, on-boarding all vehicles to the platform will require a high transactions rate during the data collection phase. The performance of the fabric network must be discussed in terms of both transaction throughput (how many transactions can go through per second) and latency (the time between sending the transaction and the finalisation of the transaction).

For the purposes of the specific system, latency is not very critical, as the data committed to the data collection chaincodes are only retrieved at a later stage, if at all. Even additional latency, which may be added because of unreliable vehicle network connections, will not create issues.

On the other hand, transaction throughput is very critical. The blockchain systems’ main performance bottleneck is the rate at which new transactions can be committed and appended to the ledger. For the presented system, the submitted transactions arrival rate depends on the number of vehicles moving around. When this surpasses the actual transaction rate that the fabric network will be able to process, a queue of pending transactions is going to be created, and its size may gradually become an issue that will hinder the system from providing the designed functionality. As has been shown by recent bench-marking efforts [32,33,34], optimized Hyperledger fabric networks can handle approximately 1000 txs/s. This means that, according to several public sources, 1000 vehicles can be served without problems, even if they continuously submit data every second. In the simulation that follows, we have taken for granted that a single Hyperledger fabric network can support up to 1000 vehicles, as is concluded by the literature. We have tested that this transaction rate is feasible in a lab environment, but we have opted not to implement the required number of installations for the experiments because of the excessive hardware requirements. We have used this metric in order to approximately map the transaction rate that must be served, in each scenario, to a number of Hyperledger fabric installations.

Data collection chaincodes hold all monitored data for all vehicles, but such data is not directly retrieved from those to be stored for incident contracts. When required, the authority organisation will manually fetch data from two or more data chaincodes to populate the incident chaincode. As no direct connection is required, more than one instance of Hyperledger fabric installations may be used to provide the appropriate transaction rate for a large smart city environment. This can either be static (by maintaining multiple Hyperledger fabric instances and permanently assigning each vehicle to a specific one) or dynamic (through a load balancing mechanism, which assigns vehicles to installations for a specific time duration). This would allow for better resources management.

When an incident happens, it will probably be required to fetch data from different fabric installations to populate the incident chaincode, but this will create minimal overhead. In terms of security, the use of multiple blockchain installations does not raise significant concerns because of the flow of information. The vehicles’ data are kept in the data contracts, which are installed in a number of blockchain networks, each one of which guarantees the security of the corresponding data. When an incident happens, the authority has to transfer data from the data contracts of the involved vehicles to the incident contract. The validity of the data transfer can be easily checked by vehicle owners who can raise a dispute in case of malicious authority activity. After that point, the security of data of the incident contracts is guaranteed by the blockchain network to which it is deployed. The immutability of every single installation is not affected by the existence of additional fabric installations, and that holds true for all the privacy and security properties of the blockchain.

In order to assess the applicability of the proposed solution to real-world cities, an extensive simulation has been conducted. A 24-h traffic dataset for the greater urban area of the city of Cologne, Germany, was used as the basis of the simulation [35,36]. The dataset was produced by a concrete simulation process that takes into account the real road network and a realistic traffic load and has produced detailed location and velocity data for every vehicle moving in the city, with a granularity of 1 s. The dataset contains an indicative traffic load for the city of Cologne. It includes all city roads, along with the highway roads around it. It is important to highlight that the main offerings of the simulated dataset are the realistic representation of vehicle movement patterns and the distribution of vehicles on the road network. The number of cars used in the simulation was taken from an analysis conducted by Uppor et al. [35,36], and thus, it is a realistic representation for the given city.

For the purposes of the current paper, we have used detailed data for each vehicle to create a realistic load of transactions for the proposed system. The main metric to be calculated was the transaction rate required to be served, especially during rush hours when peaks in vehicle commuting volume through urban areas are expected.

**Figure 8:**
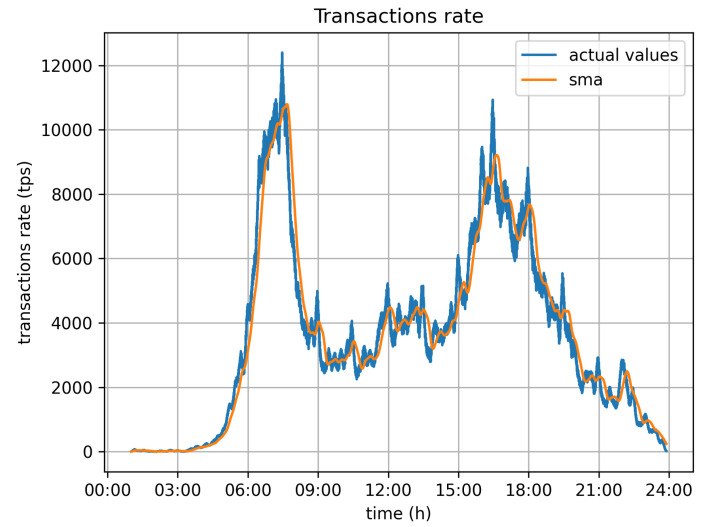
Transactions rate (initial).

Figure 8 depicts the required transaction rate to serve all vehicles moving around the city during the 24 h time window of the simulation. The numbers have been calculated on the assumption that all vehicles report data for every second. As was expected, there are two peaks in the required transaction rate, which relate to the two rush hours, early in the morning (06:00–08:00) and late in the afternoon (15:00–18:00). Those are periods during which the system may be stressed with transaction rates up to 12,000 tr/s. The blue line in the graph depicts the actual values, while the yellow one depicts the moving average (sma) of calculated values to better depict the demand.

In order to reduce the transaction rate demand, the proposed system was based on an alternative approach to the continuous record submission by vehicles. For significant parts of its course, a vehicle maintains stable direction and velocity. During those parts, it is feasible for the system to accurately estimate the vehicle’s position, even if it is not submitted.

**Figure 9:**
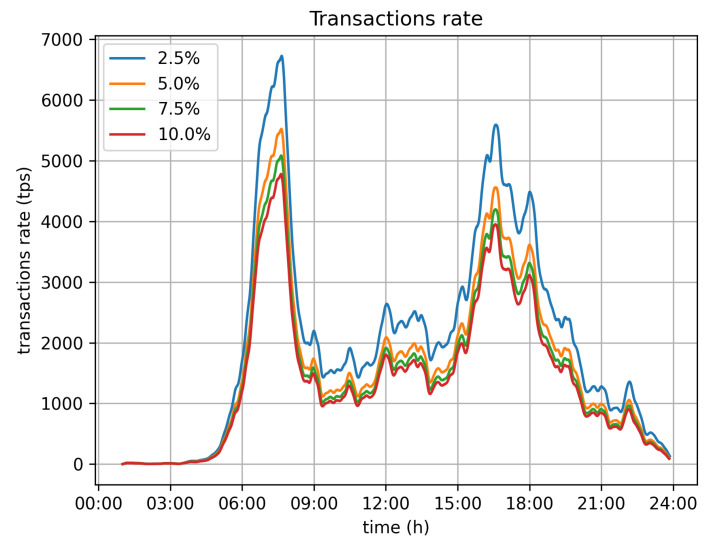
Transaction rate.

Vehicles monitor their course, and if the velocity does not deviate significantly from the average velocity of the previous five seconds, the current location submission is omitted. If the data records for such points in time are required to be retrieved, then the missing values are estimated by the previously submitted ones. The deviation limit under which this happens may vary. Figure 9 depicts the sma of the transaction rate values for different velocity deviation limits (2.5%, 5%, 7.5% and 10%). It is obvious that this approach produces significant gains with respect to transactions rate, as even with the smallest limit value, 2.5%, the transaction rate’s peak size is vastly reduced.

**Figure 10:**
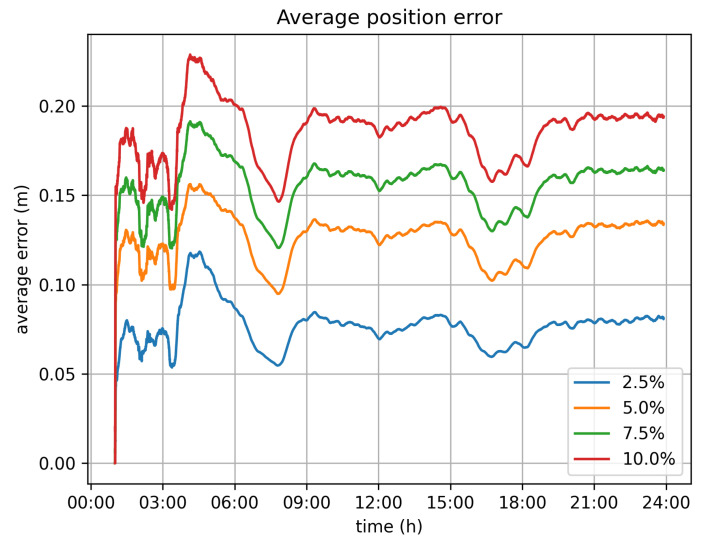
Average position error.

**Figure 11:**
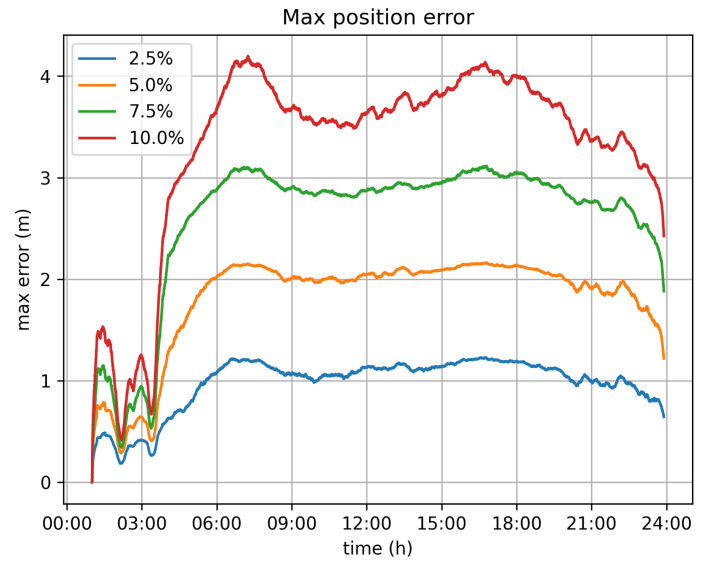
Maximum position error.

Of course, when the alternative approach of dynamically omitting location submissions is used, the estimated location records deviate from the real ones. Through the conducted simulation, we aimed to estimate the magnitude of this deviation. Figure 10 and Figure 11 depict the maximum and average error for all vehicles per timeslot. In general, the average error is rather limited and is less than 0.25 m, even for the 10% velocity limit case. The maximum error, which is the largest error value amongst all vehicles for each second is up to 4 m for the 10% velocity limit case.

With respect to the optimal value for the velocity limit parameter, the 5% option seems to be the better one. It significantly reduces the transaction rate from 11,500 tr/s to 5500 tr/s. Because of this, it demands far less infrastructure for the operation of the system. It can be efficiently served by 6 Hyperledger fabric installations (based on the assumption of 1000 tr/s for each fabric network), instead of the 12 that would be required if all values were submitted. The position error, in this case, is bearable, as the average error barely exceeds 0.15 m, while the maximum error values observed are around 2 m.

**Figure 12:**
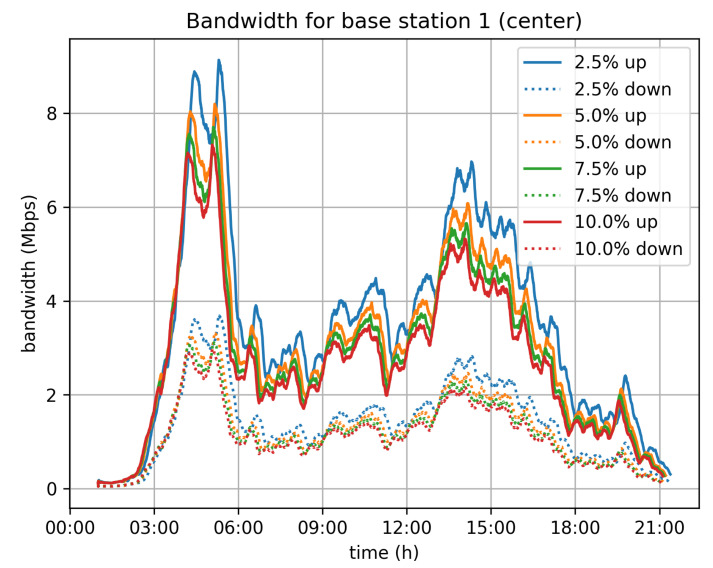
City center base station bandwidth requirements.

**Figure 13:**
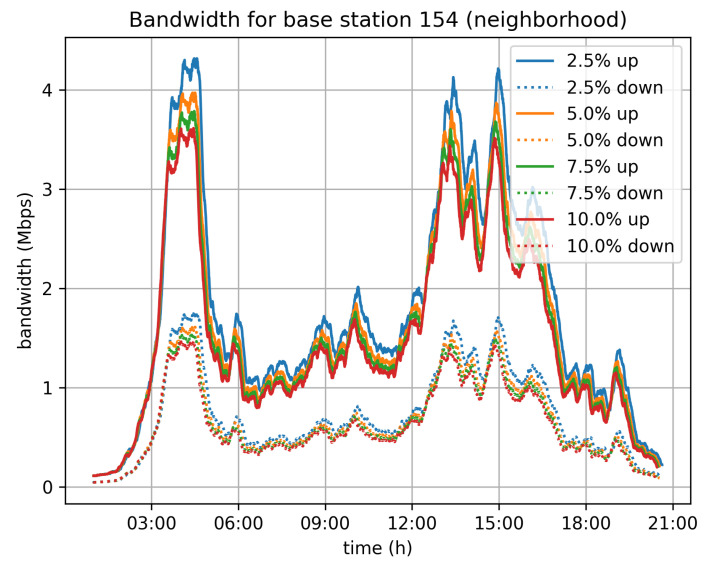
Neighborhood base station bandwidth requirements.

**Figure 14:**
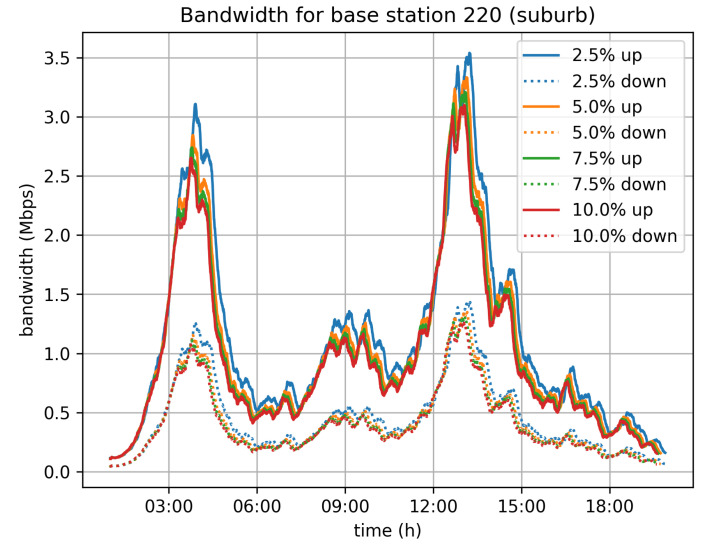
Suburbs center base station bandwidth requirements.

On top of that, we have also estimated the network traffic that will be generated by the system. Given the assumption that vehicles are connected to mobile operators’ networks, we have studied the traffic load induced for each of the cellular base stations in Cologne. We have taken for granted that each vehicle is connected to its nearest base station. This assumption may not be completely accurate, but it efficiently approximates the real distribution of the vehicles to base stations and consequently the distribution of traffic to the deployed base stations. Figure 12, Figure 13 and Figure 14 depict the bandwidth requirements for three different base stations in Cologne, located in the city center, in a neighborhood, and in the suburbs, respectively. The traffic load varies according to the traffic in the area covered by the base station. For the 5% velocity limit case, and for the city center base station, the demand peaks at 8 Mbps download and 3 Mbps upload, during the morning rush hour. For the neighborhood and base stations, the corresponding peaks are at 4 Mbps for download and 1.5 Mbps upload, while for the suburbs base station the peak of the day is observed during afternoon rush hour and the corresponding rates are 3 Mbps for download and 1 Mbps for upload.

In general, the bandwidth overhead for the base stations is not significant and the system is not expected to create any network issues, at least for a city of the size of Cologne.

**Figure 15:**
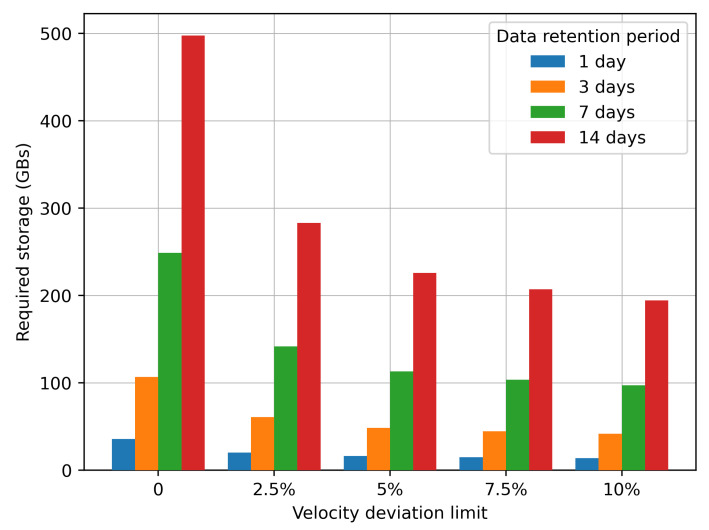
Data storage requirements.

Finally, the storage requirements for the data collected have been calculated for the case of Cologne city. It is obvious that for a city of a different size, such requirements will be different. Each transaction corresponds to an encrypted single data point of the form depicted in Equation (1), and the size of each data point is 128 bits, as it has been encrypted under the AES256 encryption algorithm. Another parameter upon which storage requirements depend is the data retention period. If authorities require participants to retain data for longer periods, then the actual storage requirements are going to be higher. In Figure 15, the storage requirements for the city of Cologne are depicted for the five different cases velocity deviation limits and four different retention periods; 1, 3, 7, and 14 days.

The results depicted in Figure 15 show that even in the less demanding case, the velocity deviation limit is set at 10% and data is kept only for 1 day, the data storage requirement is approximately 13.9 GB, which is rather high. Taken for granted the optimal value of velocity deviation limit of 5% and setting the retention period to 7 days (in order to allow for investigating incidents of the past week) the required data storage is 112.9 GBs. It is obvious that the data storage requirements are high and will be much higher for a larger city, so the use of IPFS as data storage infrastructure is a valid decision.

## 8. Conclusions

A decentralized framework to support the semi-automatic, secure settlement of vehicular incidents has been proposed. The system is based on a Hyperledger fabric and an IPFS, and supports the secure and safe storage of position and velocity data of vehicles in a smart city environment. It facilitates an easier and fairer workflow during the process of assuming or assigning liability for the incident. The system could also be used for other forensics purposes (e.g., tracking the path of a vehicle) under the strong precondition that a set of authority organisations agree on the fact that the proposed purposes are sufficient, to give access to the vehicle’s data.

The system enables the aforementioned functionality without violating the vehicle owners’ privacy. The data submitted by the vehicles remain strongly encrypted and can only be accessed either by the vehicle owner, or by an authority organisation, which is supported by the majority of other authority organisations in the system. The collection of vehicle data (velocity and location), both regarding the immediate moments before an incident and a span of time earlier than that, will be helpful in the process of liability assignment and in the scope of an investigation. This information can function either as a critical indicator for various violations or a blame absolving factor. Such data may not lead to complete resolution of every case but can greatly help towards that end in most of the cases.

A realistic traffic dataset, for a medium-sized city, has been used to assess the performance requirements for the proposed system under realistic loads. It has been proven that the application of the system is feasible as the infrastructure costs along with the communication overhead are bearable.

The proposed framework has been described in detail, but there are some points that will be further studied in our future works. The main one is the development of the on-vehicle device, for which the present paper has assumed functions in a reliable and efficient way. The actual hardware used has to be tested, along with any supplementary mechanisms that will be required with respect to connectivity reliability (e.g., supplementary network infrastructure on the road). On top of that, the cross-checking scheme that will be used for validating the data submissions from vehicles has to be defined and tested, as the validity of collected data is crucial for the system proposed.

The described system is the basis for constructing a general framework that can serve for a fair, fast, automated, and privacy-preserving workflow with respect to the resolution of liability assignment in vehicular incidents. Such a system would greatly benefit all involved actors, and will be a necessity in the near future with the uprising adoption of autonomous vehicles.

## Data Availability

In this paper we analysed a vehicular mobility dataset made available by the TAPASCologne project aimed at reproducing, with the highest level of realism possible, car traffic in the greater urban area of the city of Cologne, Germany. The dataset is available at http://kolntrace.project.citi-lab.fr/.
